# Cost of goods sold and total cost of delivery for oral and parenteral vaccine packaging formats

**DOI:** 10.1016/j.vaccine.2018.01.011

**Published:** 2018-03-14

**Authors:** Jeff Sedita, Stefanie Perrella, Matt Morio, Michael Berbari, Jui-Shan Hsu, Eugene Saxon, Courtney Jarrahian, Annie Rein-Weston, Darin Zehrung

**Affiliations:** a PATH, PO Box 900922, Seattle, WA 98109, USA; b Duff & Phelps, LLC, 55 East 52nd Street, Floor 31, New York, NY 10055, USA

**Keywords:** Vaccine, Manufacturing cost, Cost of delivery, Packaging, Rotavirus, Poliovirus, BFS, blow-fill-seal, COGS, cost of goods sold, CMYP, comprehensive multi-year plan, CPAD, compact prefilled auto-disable, DTP, diphtheria, tetanus, pertussis, FDA, U.S. Food & Drug Administration, HepB, hepatitis B, Hib, *Haemophilus influenza* type B, IPV, inactivated polio vaccine, MDV, multi-dose vial, MMD, multi-monodose, SDV, single-dose vial, TCOD, total cost of delivery, UNICEF, United Nations Children's Fund, US, United States, VVM, vaccine vial monitor

## Abstract

Despite limitations of glass packaging for vaccines, the industry has been slow to implement alternative formats. Polymer containers may address many of these limitations, such as breakage and delamination. However, the ability of polymer containers to achieve cost of goods sold (COGS) and total cost of delivery (TCOD) competitive with that of glass containers is unclear, especially for cost-sensitive low- and lower-middle-income countries.

COGS and TCOD models for oral and parenteral vaccine packaging formats were developed based on information from subject matter experts, published literature, and Kenya’s comprehensive multiyear plan for immunization. Rotavirus and inactivated poliovirus vaccines (IPV) were used as representative examples of oral and parenteral vaccines, respectively. Packaging technologies evaluated included glass vials, blow-fill-seal (BFS) containers, preformed polymer containers, and compact prefilled auto-disable (CPAD) devices in both BFS and preformed formats.

For oral vaccine packaging, BFS multi-monodose (MMD) ampoules were the least expensive format, with a COGS of $0.12 per dose. In comparison, oral single-dose glass vials had a COGS of $0.40. BFS MMD ampoules had the lowest TCOD of oral vaccine containers at $1.19 per dose delivered, and ten-dose glass vials had a TCOD of $1.61 per dose delivered. For parenteral vaccines, the lowest COGS was achieved with ten-dose glass vials at $0.22 per dose. In contrast, preformed CPAD devices had the highest COGS at $0.60 per dose. Ten-dose glass vials achieved the lowest TCOD of the parenteral vaccine formats at $1.56 per dose delivered. Of the polymer containers for parenteral vaccines, BFS MMD ampoules achieved the lowest TCOD at $1.89 per dose delivered, whereas preformed CPAD devices remained the most expensive format, at $2.25 per dose delivered.

Given their potential to address the limitations of glass and reduce COGS and TCOD, polymer containers deserve further consideration as alternative approaches for vaccine packaging.

## 1 Introduction

Historically, most vaccines have been packaged in glass containers. While the fill-finish process for vaccines in pharmaceutical-grade glass vials is well established, these containers pose a number of challenges, including breakage and delamination (flaking), which can affect product safety and efficacy [1], [2], [3]; programmatic wastage of vaccines lacking preservatives and packaged in multidose-vials [4]; appropriate disposal in low-resource settings [5], [6]; and the cost per dose of manufacturing for single-dose vials relative to multidose-vials. Alternative packaging formats—including polymer containers—are increasingly used, both for oral and parenteral pharmaceuticals, and they may address some of the limitations of glass-based packaging. However, the ability of polymer containers to achieve a cost of goods sold (COGS) and total cost of delivery (TCOD) competitive with that of glass containers is unclear, especially for cost-sensitive low- and lower-middle-income countries.

Two polymer fill-finish approaches are preformed (injection-molded) polymer containers and blow-fill-seal (BFS) packaging. These containers can be formed from a variety of polymers based on the preferred container characteristics and vaccine or pharmaceutical compatibility [7]. Preformed containers are purchased as sterile, open containers from vendors, filled with the biopharmaceutical, and sealed under sterile conditions. BFS containers are formed in a continuous process during which melted resin is extruded, blown into molds, formed, filled with biopharmaceutical, and sealed within a matter of seconds [8], [9], [10].

Both preformed polymer containers and BFS packaging enable a broad array of designs, including some in which the primary packaging also serves as the delivery device. For oral delivery, the primary container can be opened and contents dispensed directly into the patient’s mouth; e.g., currently marketed rotavirus vaccines [11], [12]. Polymer containers can be manufactured as ampoules, as well as compact prefilled auto-disable (CPAD) devices, which can include an integrated needle; e.g., the Uniject™ CPAD injection system [13]. Such devices can simplify delivery, ensure the correct dose is administered, and prevent transmission of blood-borne infections associated with needle reuse [14], [15]. Polymer containers also enable multi-monodose (MMD) designs—multiple single-dose containers conjoined by a shared tab with one vaccine vial monitor and product label affixed to the tab [16], [17]. MMD designs could reduce manufacturing cost and cold chain volume compared with traditional glass vial packaging.

While other studies have considered the potential cost of vaccine manufacturing for the developing world or compared the cost of vaccine administration for a single polymer container with that of glass vials, no studies have compared both the fill-finish cost and the total cost of delivery across a variety of alternative packaging formats with those of single- and multi-dose glass vials [18], [19].

The aim of our study was to quantify the economic differences among vaccine presentations for low- and lower-middle-income country markets, as defined by the World Bank [20], by evaluating the COGS from a manufacturing perspective and the TCOD from a programmatic perspective for glass vials, preformed polymer containers, and BFS packaging. In addition, we considered a number of prototype packaging formats, which may help establish an evidence base to support efforts to implement these technologies as vaccine packaging formats. Our model used IPV and rotavirus vaccine as representative examples of parenteral and oral vaccines, respectively.

## 2 Methodology

**Fig. 1:**
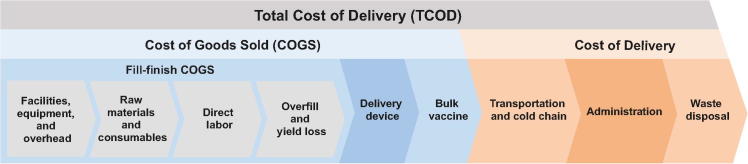
Depiction of the model flow and boundaries of each model.

The overall modeling flow is shown in Fig. 1. To create a useful comparison, the analysis estimates costs on an annual basis over a period of steady production at equivalent volumes for each presentation.

### 2.1 Cost of goods sold

**Fig. 2:**
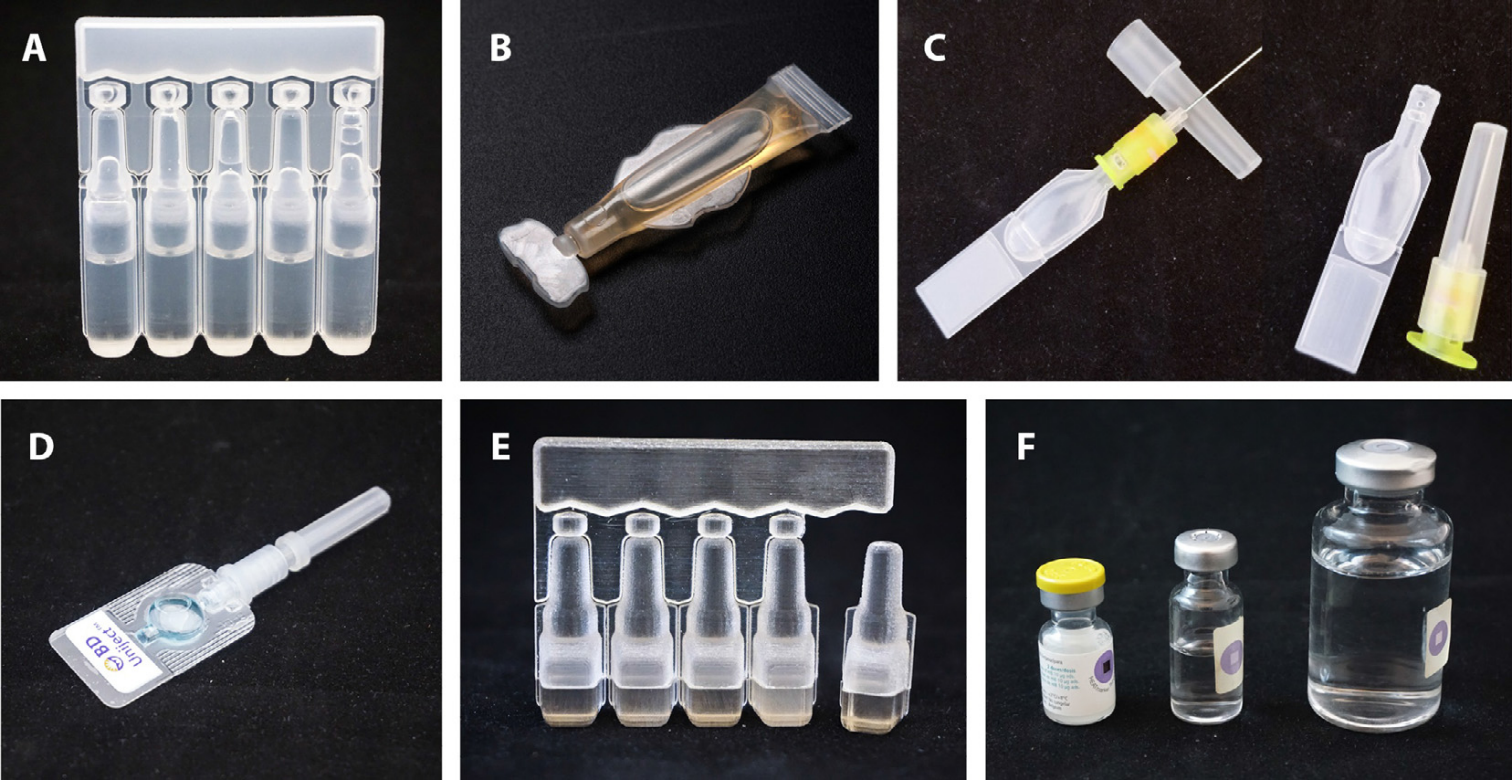
Representative examples of the devices evaluated in the cost of goods sold and total cost of delivery models. (A) Oral BFS MMD ampoule; (B) preformed polymer tube; (C) BFS CPAD devices with and without separately packaged needle attached (not shown: BFS CPAD device prior to removal from five-dose card); (D) preformed CPAD device (Uniject™ injection system shown as a representative example); (E) parenteral BFS MMD ampoule (image shown is a three-dimensional printed prototype); and (F) glass vials (left to right: 2R/31 mm single-dose vial for oral and parenteral vaccines, 4R ten-dose vial for parenteral vaccines, and 20R ten-dose glass vial for oral vaccines). Abbreviations: BFS, blow-fill-seal; CPAD, compact prefilled auto-disable; MMD, multi-monodose.

For oral vaccine packaging presentations, we evaluated four primary containers designed for a 2 mL dose: BFS MMD ampoules, preformed polymer tubes, a single-dose glass vial, and a ten-dose (20 mL) glass vial (Fig. 2). The BFS MMD device consisted of five single-dose ampoules joined by a tab. The preformed polymer tubes were packaged as single-dose, individually labeled tubes.

For parenteral vaccine packaging presentations, we evaluated five primary containers designed for a 0.5 mL dose: BFS MMD ampoules that require a separate needle and syringe for delivery, a BFS CPAD device of an MMD design with a separately packaged custom needle assembly, a preformed polymer CPAD device with an integrated needle, a single-dose glass vial, and a ten-dose (5 mL) glass vial (Fig. 2).

The BFS containers were prototype designs with features anticipated to be required for regulatory approval, such as sufficient labeling space; however, none of these has yet been used as primary packaging for vaccines. Preformed polymer tubes and preformed CPAD devices are commercially available.

Secondary packaging for each oral and parenteral container design was optimized to reduce cold chain volume. It was assumed that parenteral vaccines in polymer packaging required overwrap—given the potential impact of gas exchange on the small volume—but that those in glass would not require overwrap. For oral presentations, no overwrap was included. All formats were packaged 50 doses per secondary package, except ten-dose glass vials, which were packaged 50 vials (500 doses) per secondary container.

Our COGS analysis—with inputs from manufacturers and industry experts—estimated postformulation through tertiary packaging costs incurred by a manufacturer, assuming an annual production volume of 50 million doses of vaccine. Fill-finish costs included the following categories:

(1) **Facilities, equipment, and overhead** included depreciation of capital expenditures (capitalized over a 20-year and 10-year economic useful life for facilities and equipment, respectively) and ongoing annual overhead costs for repairs and maintenance, utilities, and indirect and corporate overhead for a dedicated 50 million annual throughput filling line in a United States (US) brownfield facility.
(2) **Raw materials** included presentation-specific primary, secondary, and tertiary packaging materials, foil overwrap (polymer parenteral presentations only), labels, and cartons (secondary and tertiary).
(3) **Direct labor** included fill line operators, fill line clearance, and packaging line operators. Costs were based on hourly labor rates and the time required to fill 50 million doses, assuming a 500 L batch size for rotavirus vaccine (2.0 mL per dose) and a 125 L batch size for IPV (0.5 mL per dose).
(4) **Overfill** was estimated at a maximum of 8% and 20% for single-dose rotavirus vaccine and IPV presentations, respectively [21]. Ten-dose glass vial overfill was estimated at 2% and 6% for rotavirus vaccine and IPV, respectively. A sensitivity analysis of overfill estimates was performed and is detailed in Section 2.3.1 below.
(5) **Yield loss** was factored in as the estimated bulk product loss during production, based on the specific filling and finishing process for each product.

We added the following costs separately from fill-finish costs to allow a clearer comparison among packaging types:

(6) **Delivery device** included the cost of a separately packaged syringe for the parenteral BFS ampoule and glass vial presentations. The costs of an oral delivery syringe (one per dose) and vial adapter (one per vial) were included for oral vial presentations.
(7) **Bulk formulation costs** were the estimated bulk vaccine formulation costs for rotavirus vaccine ($0.48 per dose or $0.24/mL) and IPV ($0.99 per dose or $1.98/mL).

Our analysis excludes product development and regulatory costs. Key inputs to the COGS model are in Table 1A and B. For a more detailed methodology of the COGS analysis, see Supplementary material, Appendix A.

**Table 1:** Cost of goods sold model: Key inputs by container type for (A) oral vaccine containers and (B) parenteral vaccine containers.

Oral vaccine containers				
Facility and equipment	BFS MMD ampoule	Preformed polymer tube	Single-dose glass vial	Ten-dose glass vial
US brownfield facility	$2,775,000	$1,711,500	$22,550,000	$22,550,000
Cold storage buildout	$864,482	$1,232,346	$735,729	$441,438
Peripheral equipment (constant)	$1,900,000	$1,900,000	$1,900,000	$1,900,000
Manufacturing equipment	$8,231,306	$5,755,000	$8,065,000	$8,065,000
Total facility and equipment CAPEX	$13,770,788	$10,598,846	$33,250,729	$32,956,438

*Overhead*				
Repairs & maintenance	$383,922	$283,663	$707,929	$707,929
Utilities	$400,000	$400,000	$600,000	$500,000
Indirect labor & corporate overhead (constant)	$400,000	$400,000	$400,000	$400,000
Total annual ongoing overhead costs	$1,183,922	$1,083,663	$1,707,929	$1,607,929

*Raw materials and consumables*				
Total primary raw material cost (low estimate)	$150,000	$2,000,000	$6,000,000	$750,000
Total primary raw material cost (average)	$200,000	$2,500,000	$7,500,000	$925,000
Total primary raw material cost (high estimate)	$250,000	$3,000,000	$9,000,000	$1,100,000
Total secondary raw materials (low estimate)	$672,881	$2,737,288	$2,700,000	$317,797
Total secondary raw material cost (average)	$794,492	$3,266,949	$3,225,000	$376,271
Total secondary raw materials (high estimate)	$916,102	$3,796,610	$3,750,000	$434,746
Total annual raw materials (average)	$994,492	$5,766,949	$10,725,000	$1,301,271

*Direct Labor*				
Filling	$388,385	$1,242,834	$1,670,079	$167,008
Line clearance	$53,187	$132,967	$428,824	$404,884
Packaging	$125,000	$125,000	$125,000	$12,500
Total annual direct labor costs	$566,572	$1,500,800	$2,223,903	$584,392

*Overfill and yield loss*				
Drug dosage (mL) (constant)	2.0	2.0	2.0	2.0
Dosage per presentation (mL) (constant)	2.0	2.0	2.0	20.0
Yield loss cost per dose	$0.00	$0.00	$0.02	$0.02
Overfill cost	$0.04	$0.04	$0.04	$0.01
Overfill and yield loss per dose	$0.04	$0.04	$0.06	$0.03

*Delivery device*				
Delivery device cost per dose	$0.00	$0.00	$0.12	$0.15

*Vaccine*				
Vaccine cost per dose (constant)	$0.48	$0.48	$0.48	$0.48

Parenteral vaccine containers					
Facility and equipment	BFS MMD ampoule	BFS CPAD device	Preformed CPAD device	Single-dose glass vial	Ten-dose glass vial

US brownfield facility	$2,775,000	$2,775,000	$2,250,000	$22,550,000	$22,550,000
Cold storage buildout	$717,336	$698,943	$965,645	$809,302	$110,359
Peripheral equipment (constant)	$1,900,000	$1,900,000	$1,900,000	$1,900,000	$1,900,000
Manufacturing equipment	$8,631,306	$8,631,306	$5,865,000	$8,065,000	$8,065,000
Total facility and equipment CAPEX	$14,023,642	$14,005,249	$10,980,645	$33,324,302	$32,625,359

*Overhead*					
Repairs & maintenance	$397,254	$397,254	$296,304	$707,929	$707,929
Utilities	$400,000	$500,000	$400,000	$600,000	$500,000
Indirect labor & corporate overhead (constant)	$400,000	$400,000	$400,000	$400,000	$400,000
Total annual ongoing overhead costs	$1,197,254	$1,297,254	$1,096,304	$1,707,929	$1,607,929

*Raw materials and consumables*					
Total primary raw material cost (low estimate)	$150,000	$450,000	$7,500,000	$6,000,000	750,000
Total primary raw material cost (average)	$200,000	$600,000	$10,000,000	$7,500,000	925,000
Total primary raw material cost (high estimate)	$250,000	$750,000	$12,500,000	$9,000,000	$1,100,000
Total secondary raw materials (low estimate)	$1,902,542	$1,892,373	$4,238,771	$2,689,831	$256,780
Total secondary raw material cost (average)	$2,646,610	$2,635,169	$5,542,055	$3,213,559	$307,627
Total secondary raw materials (high estimate)	$3,390,678	$3,377,966	$6,845,339	$3,737,288	$358,475
Total annual raw materials (average)	$2,846,610	$3,235,169	$15,542,055	$10,713,559	$1,232,627

*Direct labor*					
Filling	$388,385	$417,520	$1,553,542	$2,004,095	$200,409
Line clearance	$59,370	$159,559	$296,851	$478,678	$422,913
Packaging	$166,667	$166,667	$166,667	$125,000	$12,500
Total annual direct labor costs	$614,422	$743,746	$2,017,059	$2,607,773	$635,823

*Overfill and yield loss*					
Drug dosage (mL) (constant)	0.5	0.5	0.5	0.5	0.5
Dosage per presentation (mL) (constant)	0.5	0.5	0.5	0.5	5.0
Yield loss cost per dose	$0.01	$0.05	$0.01	$0.05	$0.05
Overfill cost	$0.20	$0.20	$0.20	$0.20	$0.06
Overfill and yield loss per dose	$0.21	$0.25	$0.21	$0.25	$0.11

*Delivery device*					
Delivery device cost per dose	$0.04	$0.04	$0.00	$0.04	$0.04

*Vaccine*					
Vaccine cost per dose (constant)	$0.99	$0.99	$0.99	$0.99	$0.99

Abbreviations: BFS, blow-fill-seal; CAPEX, capital expenditure; COGS, cost of goods sold; CPAD, compact prefilled auto-disable; MMD, multi-monodose.

### 2.2 Total cost of delivery

The outputs from the COGS analysis, which excluded profit margin and were not representative of actual purchase prices for the vaccines, served as inputs into the cost of delivery model. We estimated the TCOD for one year and one birth cohort from the point of receipt in country through the point of immunization delivery, using Kenya as a model country based on data from Kenya’s comprehensive multiyear plan for immunization [22], [23], drawing from country data [24], PATH models [25], and published material [26]. Key inputs for the TCOD model are in Table 2. TCOD cost categories included the following:

(1) **Vaccine cost** was the output of the COGS analysis multiplied by the number of doses required to vaccinate the target population in a year, including programmatic wastage. For oral rotavirus vaccine, which lacks preservatives, wastage rates were assumed to be 50% for ten-dose glass vials and 5% for single-dose presentations (PATH estimates). For IPV, which contains preservatives, wastage rates were assumed to be 15% for ten-dose glass vials and 5% for single-dose presentations (PATH estimates).
(2) **Transportation and cold chain storage.** Trucks, fuel, cold boxes, and average distance between cold chain facilities were used to cost transport of vaccines. Storage costs were calculated per liter of storage, including maintenance at each level, based on existing equipment. Human resource costs were not included, as labor costs are not accretive to the analysis at a per-dose level.
(3) **Administration** included the cost of a health care worker’s time for vaccine administration in the routine immunization setting (PATH estimates). We based times for novel delivery technologies on target product profile guidance documents and correlation with previously conducted studies [27].
(4) **Waste disposal** included the cost of disposing the primary packaging and delivery device (if required) on a volume-per-dose basis [28].

**Table 2:** Total cost of delivery model - key inputs by container type. Packaged volume was calculated as the total volume of the secondary packaging (including foil overwrap for the blow-fill-seal multi-monodose ampoule, blow-fill-seal compact prefilled auto-disable device, and preformed compact prefilled auto-disable for parenteral vaccines) divided by the number of doses per secondary package.

	Oral vaccine containers				Parenteral vaccine containers				
	BFS MMD ampoule	Preformed polymer tube	Single-dose glass vial	Ten-dose glass vial	BFS MMD ampoule	BFS CPAD device	Preformed CPAD device	Single-dose glass vial	Ten-dose glass vial
Packaged volume/dose (incl 2° packaging)	9.4 cm^3^	13.4 cm^3^	8.0 cm^3^	4.8 cm^3^	7.8 cm^3^	7.6 cm^3^	10.5 cm^3^	8.0 cm^3^	1.1 cm^3^
Delivery device volume	–	–	36.5 cm^3^	31.1 cm^3^	42.8 cm^3^	16 cm^3^	–	42.8 cm^3^	42.8 cm^3^
Waste disposal volume/dose	9.4 cm^3^	13.4 cm^3^	44.5 cm^3^	35.9 cm^3^	50.6 cm^3^	23.6 cm^3^	10.5 cm^3^	50.8 cm^3^	43.9 cm^3^
Wastage rate	5%	5%	5%	50%	5%	5%	5%	5%	15%

Abbreviations: BFS, blow-fill-seal; CPAD, compact prefilled auto-disable; MMD, multi-monodose.

For a more detailed methodology of the TCOD analysis, see the Supplementary material, Appendix B.

### 2.3 Sensitivity analysis

To understand the impact of variability in key cost drivers related to design of polymer container formats, a sensitivity analysis was performed on overfill and packaging volume.

#### 2.3.1 COGS sensitivity

(1) **Overfill.** Based a dose expression study of sample prefilled polymer containers (data not shown), a reduced overfill percentage of 5% for oral containers, 11% for BFS CPAD, and 12% for preformed CPADs were assessed. Overfill for vials and ampoules intended for injection was based on the USP guidance, which serves as a U.S. Food & Drug Administration (FDA) requirement, and was not varied [29].

#### 2.3.2 TCOD sensitivity

(1) **Vaccine cost.** The change in vaccine cost due to variations in overfill from the COGS sensitivity analysis was assessed to understand the impact on the TCOD.
(2) **Packaged volume.** For the polymer containers, we assessed the impact of doubling the packaged volume per dose to account for potential variability in primary and secondary container designs. Given the standardized guidances for glass vials, we assumed no change in packaged volume per dose for glass vials and separate delivery devices.

## 3 Results

All results are presented on a cost per dose basis.

### 3.1 Fill-finish cost of goods sold

**Fig. 3:**
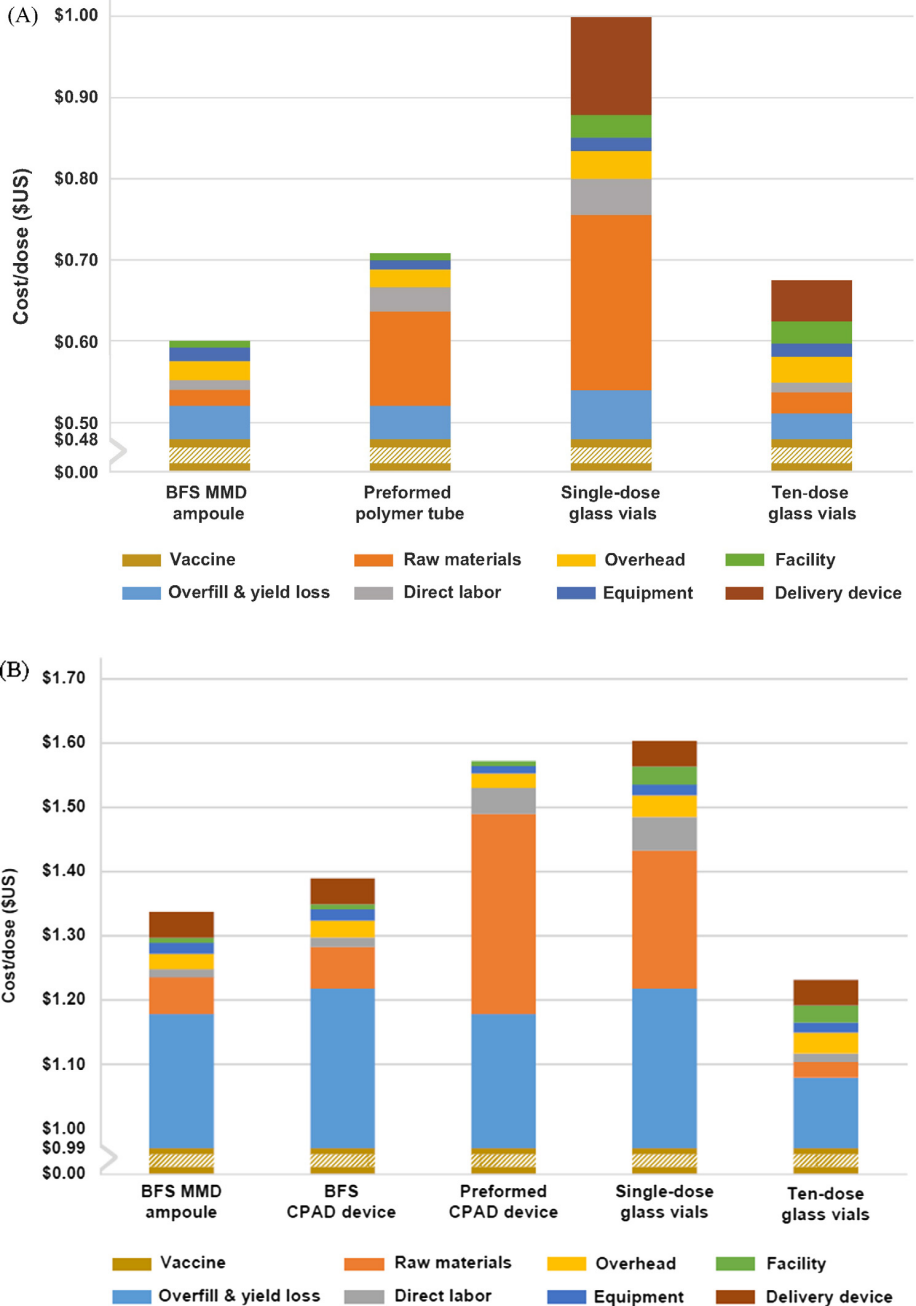
Cost of goods sold for (A) oral and (B) parenteral vaccine containers with a breakdown of cost by category. Values shown on a cost per dose basis in US dollars ($). Fill-finish costs included everything except the vaccine and delivery device. Abbreviations: BFS, blow-fill-seal; CPAD, compact prefilled auto-disable; MMD, multi-monodose.

The fill-finish COGS did not include costs associated with bulk formulation cost (other than overfill and yield loss) or separately packaged/purchased delivery devices. For oral packaging formats, single-dose glass vials were the most expensive format, with a fill-finish cost of $0.40 each. In contrast, the fill-finish cost for BFS MMD ampoules, ten-dose glass vials, and preformed polymer tubes were $0.12, $0.14, and $0.23, respectively, representing a 70–43% reduction compared with single-dose glass vials (Fig. 3A).

For parenteral vaccine containers, preformed CPAD devices were the most expensive packaging format, with a fill-finish cost of $0.60. The fill-finish cost for ten-dose glass vials, BFS MMD ampoules, BFS CPAD devices, and single-dose glass vials was $0.22, $0.33, $0.38, and $0.59, respectively, representing a reduction of up to 63% (Fig. 3B).

### 3.2 Fill-finish cost of goods sold cost drivers

For both parenteral and oral vaccine containers, the key categories driving fill-finish COGS were raw materials and consumables, as well as overfill and yield loss (Fig. 3A and B). For oral containers, raw materials and consumables represented 17% of fill-finish COGS for BFS ampoules, 51% for preformed polymer tubes, 54% for single-dose glass vials, and 18% for ten-dose glass vials. Compared with single-dose glass vials, raw materials and consumables accounted for 70% and 75% of the savings achieved by BFS MMD ampoules and ten-dose glass vials, respectively.

For parenteral containers, raw materials and consumables represented 17% of fill-finish COGS for BFS ampoules, 17% for BFS CPAD devices, 52% for preformed CPAD devices, 36% for single-dose glass vials, and 11% for ten-dose glass vials, respectively. Raw materials alone accounted for 59% and 70% of the savings afforded by the BFS MMD ampoules and BFS CPAD devices, respectively, compared with single-dose glass vials.

Collectively, the depreciated facility and equipment costs and annual overhead contributed between $0.04 and $0.08 per dose to fill-finish COGS for both oral and parenteral vaccine presentations. Direct labor was $0.01 for all BFS presentations and ten-dose glass vials. Direct labor ranged from $0.03 to $0.05 for all other presentations and did not exceed 13% of fill-finish COGS for any presentation.

Overfill and yield loss accounted for $0.03–0.06 of the total fill-finish COGS for oral presentations and $0.11–0.25 for parenteral presentations, reflecting the difference in the cost of bulk formulation.

### 3.3 Total cost of goods sold

Single-dose glass vials were the most expensive packaging format for oral rotavirus vaccine, at $1.00 per dose, with the remaining vaccine packaging formats ranging from $0.60 for BFS MMD ampoules to $0.71 for the preformed polymer tubes (Fig. 3A) when the cost of bulk formulation and delivery device were considered in addition to fill-finish COGS.

Single-dose glass vials were also the most expensive packaging format for parenteral IPV, at $1.62 per dose, with the remaining vaccine packaging formats ranging from $1.25 for ten-dose glass vials to $1.59 for the preformed polymer CPAD device (Fig. 3B) when the cost of bulk formulation and delivery device were included.

### 3.4 Total cost of delivery

**Fig. 4:**
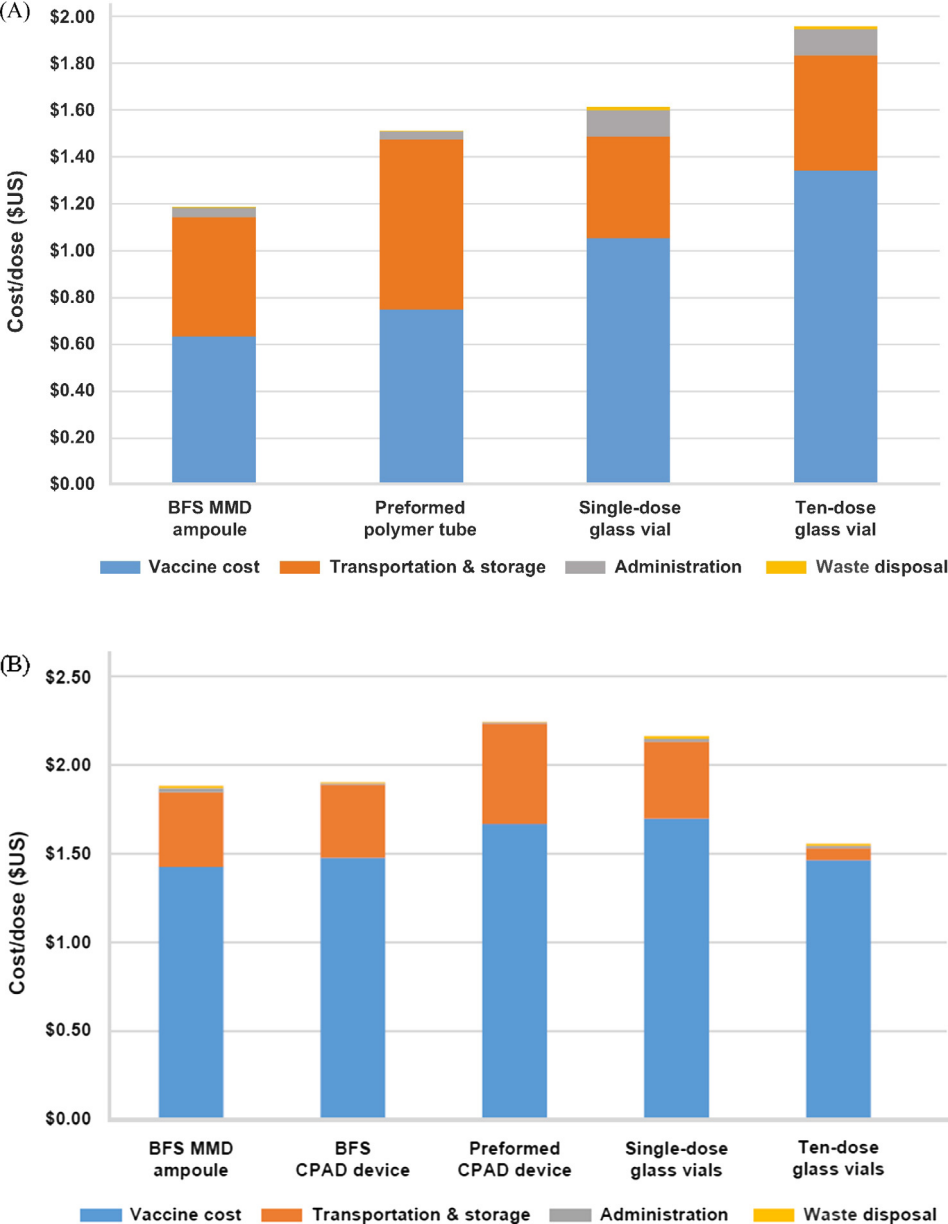
Total cost of delivery for (A) oral rotavirus vaccine and (B) parenteral IPV for routine immunization in Kenya. Values shown on a cost per dose basis in US dollars ($). ^*^Vaccine cost was the output of the COGS analysis, including programmatic wastage. Abbreviations: BFS, blow-fill-seal; CPAD, compact prefilled auto-disable; MMD, multi-monodose.

The estimated total cost per dose of oral rotavirus vaccine delivered was highest for ten-dose glass vials, at $1.96, and lowest for BFS MMD ampoules, at $1.19 (Fig. 4A). For parenteral IPV, the TCOD was highest for the preformed CPAD device, at $2.25, and lowest for the ten-dose glass vial, at $1.56 (Fig. 4B).

Vaccine cost (the output from the COGS analysis) for rotavirus vaccine was 49% of the TCOD for BFS MMD ampoules and 68% for ten-dose glass vials (Fig. 4A). For IPV, vaccine cost was 74% of the TCOD for the preformed CPAD device and 94% for ten-dose glass vials (Fig. 4B).

Excluding vaccine cost, the cost for transportation and cold chain storage was the greatest contributor to the TCOD. For rotavirus vaccine, this cost was highest for preformed polymer tubes, at $0.73, and lowest for single-dose glass vials, at $0.43 (Fig. 4A). For IPV, the cost of transportation and cold chain storage was $0.57 for preformed CPAD devices and $0.07 for ten-dose glass vials (Fig. 4B). Although the ten-dose glass vial for rotavirus had smaller dimensions than other oral containers, its greater programmatic wastage (50% versus 5%), a reflection of the lack of preservatives, resulted in a greater transportation and cold chain costs compared with that of single-dose glass vials (Table 2).

The cost of administration for oral presentations was $0.11 for the single-dose glass vial and ten-dose glass vial. In contrast, administration costs were $0.04 for the preformed polymer tube and $0.03 for the oral BFS MMD ampoule (Fig. 4A). The contribution of administration cost to the TCOD for parenteral presentations was $0.02 for BFS MMD ampoules and single-dose glass vials, and $0.01 for BFS CPAD devices, preformed CPAD devices, and ten-dose glass vials (Fig. 4B).

Waste disposal had a minimal impact on the TCOD, comprising less than $0.02 for both oral and parenteral vaccines (Fig. 4A and B).

### 3.5 Sensitivity analysis

**Fig. 5:**
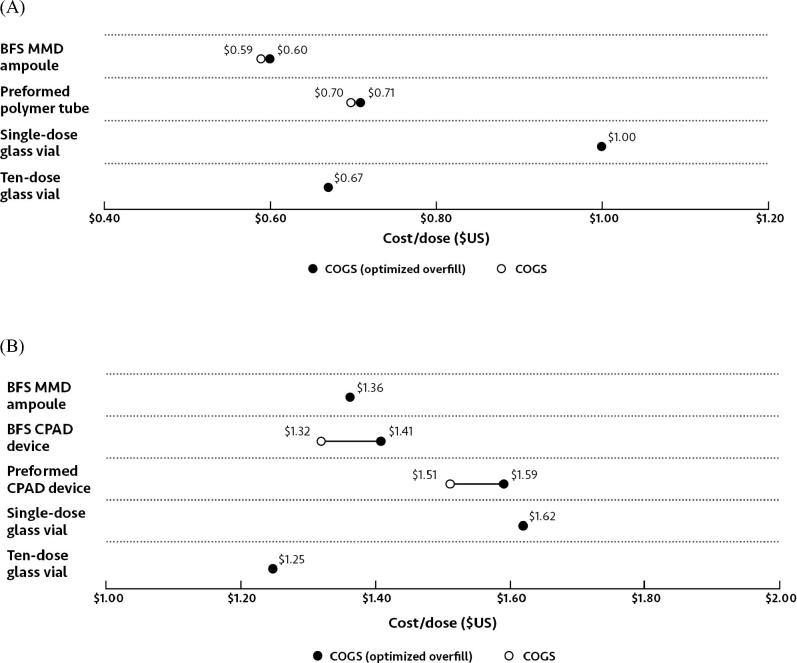
Sensitivity analysis for (A) oral and (B) parenteral vaccine packaging formats, showing the impact of variability in percentage overfill on COGS. Values shown on a cost per dose basis in US dollars. Abbreviations: BFS, blow-fill-seal; CPAD, compact prefilled auto-disable; MMD, multi-monodose; COGS, cost of goods sold.

For both oral BFS MMD ampoules and preformed plastic tubes, reducing overfill from 8% to 5% reduced COGS by $0.01 (Fig. 5A). For parenteral presentations, under the minimum overfill scenario, COGS dropped from $1.59 to $1.51 for the preformed CPAD and from $1.41 to $1.32 for the BFS CPAD, making the latter less expensive than BFS MMD ampoules (Fig. 5B).

**Fig. 6:**
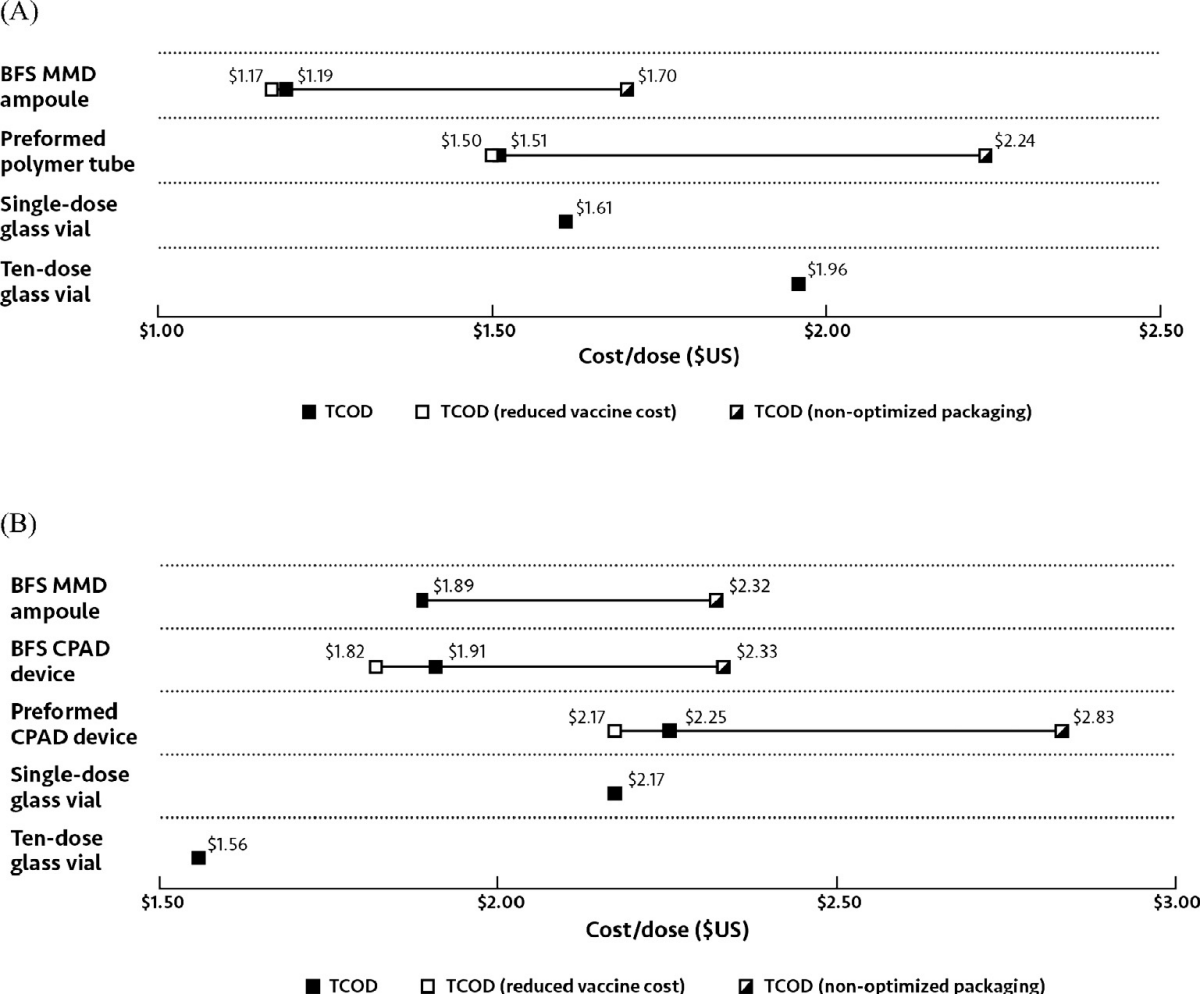
Sensitivity analysis for (A) oral and (B) parenteral vaccine packaging formats, showing the impact of variability of overfill and packaged volume on TCOD. Values shown on a cost per dose basis in US dollars ($). Abbreviations: BFS, blow-fill-seal; CPAD, compact prefilled auto-disable; MMD, multi-monodose; TCOD, total cost of delivery.

When packaged volume was doubled, the TCOD for oral packaging formats increased by $0.51 and $0.73 for BFS MMD ampoules and preformed packaged tubes, respectively (Fig. 6A). For the parenteral packaging formats, doubling packaged volume increased TCOD by $0.41 for the BFS CPAD, $0.43 for the BFS MMD ampoules, and $0.57 for the preformed CPAD (Fig. 6B). Nearly all of the increase in TCOD can be attributed to the increased cost of transportation and storage, as the impact of increased packaging volume on waste disposal was less than $0.01 for all packaging formats.

## 4 Discussion

To the best of our knowledge, the analysis documented here is the first to evaluate not only the fill-finish COGS for a variety of packaging technologies from a manufacturer’s perspective, it is also the first to add delivery-related costs, assessing the TCOD for oral (rotavirus) and parenteral (IPV) vaccines over a variety of packaging formats. For oral vaccines, polymer containers achieved lower COGS and TCOD than either single-dose or ten-dose glass vials—in part because the BFS MMD ampoules and the preformed polymer tubes eliminated the need for separate delivery devices. They also reduced programmatic wastage compared with multi-dose-vials—important for rotavirus vaccine, which does not contain preservatives.

For parenteral vaccines, BFS MMD ampoules and BFS CPAD devices achieved a lower TCOD than single-dose glass vials, but none of the polymer containers could achieve a COGS or TCOD lower than ten-dose glass vials. Several issues limit the potential of polymer containers within this context, including (1) a reduced differential in delivery device (i.e., needle and syringe) cost between polymer and glass packaging formats, compared with the differential between delivery device cost for oral packaging; (2) the requirement for foil overwrap on polymer containers, which increases both COGS and—by increasing cold chain volumes—the TCOD; and (3) the differential in overfill between parenteral single-dose presentations (20%) and ten-dose vials (6%), an impact that will be further amplified for vaccines with a high cost of bulk antigen or formulation, which is the case for IPV. These issues highlight the need to consider specific vaccine characteristics (e.g., antigen and formulation cost, use of preservatives) in addition to the fill-finish COGS when evaluating packaging formats.

The sensitivity analysis reveals the importance of optimizing overfill for those packaging formats that don’t require the dose be drawn by syringe prior to administration. Reducing overfill may be of limited benefit for vaccines with low formulation cost or large dose volumes because overfill decreases as dose volume increases. However, reducing overfill for expensive, small dose volume vaccines can have a meaningful impact in the reduction of cost for both vaccine manufacturers and potentially vaccine purchasers, assuming such cost savings are passed along.

Perhaps more important than reducing overfill is the importance of optimizing designs of primary and secondary packaging, given that a doubling in the packaged volume per dose resulted in an increased TCOD ranging from $0.41 to $0.73 per dose. In all cases, increasing the packaged volume of the polymer containers resulted in increased storage and transportation cost, making the TCOD potentially greater than that of single-dose glass vials. Optimization of the packaged volume of polymer containers could be achieved by a variety of means, including design changes that reduce the physical footprint or enable nesting of the primary containers and minimizing dead space within the secondary packaging. Optimizing or eliminating overwrap could also aid in the reduction of packaged volume for polymer containers. Depending on intended storage conditions and susceptibility to gas and water vapor permeation, some vaccines may not require overwrap. For those needing such protection, modifying polymer primary container material or incorporating barriers into more efficient secondary packaging configurations could minimize packaging volume. Optimization of overfill, container design, and secondary packaging of polymer containers should ideally be addressed during vaccine development to avoid the need for product changes post licensure, which can have cost, manufacturing, and regulatory implications.

Overall, this analysis demonstrated that polymer containers have the potential to reduce the cost of manufacturing and vaccination compared with glass vials for both oral and parenteral vaccines. In addition to the potential for reducing COGS and TCOD, polymer packaging could remove risk of delamination, reduce potential for damage during transport, and improve ease of use [30]. However, consideration should be given to design specifications and optimization of packaging layouts, given the impact that packaged volume can have on the transportation and cold chain storage cost.

While cost is one major driver of primary container selection, additional factors must be considered. Key issues include technical compatibility of the selected packaging format with the vaccine, usability and acceptability, and the logistical and programmatic impacts of introducing novel container types [31], [32], [33]. For example, certain adjuvants may be susceptible to adhering to polymer containers making them less suitable packaging solutions than glass containers [34]. In addition, the usability of the device is of critical importance to health care workers and can impact the degree of training required [35], [36]. Usability factors such as intuitiveness of use and the ability to easily squeeze and deliver vaccines from both oral and parenteral polymer packaging formats are dependent on the geometry and thickness of the container and viscosity of the vaccine [34]. Stakeholder input on the currently available Uniject™ CPAD has highlighted the simplicity, safety, and cold chain benefits of this preformed polymer container for vaccines [30], [37]. PATH has conducted formative usability and acceptability studies of novel oral and parenteral polymer containers among health care workers and immunization program managers in low-resource settings. Stakeholders identified several positive attributes, such as their intuitive use and flexibility to suit varying supply chain configurations and program implementation strategies (unpublished results).

A reduction in COGS may translate to reduced prices for low- and lower-middle-income countries; however, from the manufacturing viewpoint, switching from glass vial to a polymer packaging fill-finish line will also require significant investment in validation and regulatory approval. These investments will increase the cost of products relative to the findings from this analysis. However, the flexibility in design afforded by polymers may allow optimization of packaged volume to minimize cold chain impact, the second greatest driver of TCOD.

### 4.1 Limitations

This analysis has several limitations. We focused on two representative vaccines as examples, but we anticipate that the general trends observed could be applicable to other oral and parenteral vaccines and pharmaceuticals, although those use cases would require analysis. While manufacturing experts were consulted in the development of the models for the analysis, their information was limited because some of the technologies were still in development. Furthermore, the oral and parenteral BFS MMD ampoules and parenteral BFS CPAD device containers were prototypes; modifications to these designs—or to commercially available designs modeled—would produce different findings. In addition, the COGS model assumed manufacturing in a US-based brownfield facility, and the TCOD model used the Kenya supply chain. Variations from these scenarios would likely generate different outcomes. Finally, we did not include research and development cost, regulatory cost, or profit margin, due to the high degree of variability among manufacturers, markets, and products.

A more comprehensive analysis that includes research and development, regulatory, and changeover costs will allow fuller understanding of the financial implications of different packaging formats for vaccine manufacturers and purchasers. Given the additional capital investment required to change over from one packaging technology to another, the results of this analysis are more useful to those manufacturers considering investment in new production capacity. Nevertheless, these findings suggest that polymer packaging formats may offer reduced manufacturing cost and could enable savings for vaccination programs in low- and lower-middle-income countries.

## Disclaimer

Any opinions presented in this article are those of the author(s) and do not necessarily represent the official position of Duff & Phelps.

## Conflict of interest

PATH has received funding from rommelag in connection with projects unrelated to this manuscript. PATH licensed the Uniject™ injection system technology to BD but does not receive any financial compensation or incentive in connection with the transaction. Duff & Phelps provides professional services to a variety of companies operating within the biotech industry and is compensated for such services. The professional services are unrelated to this manuscript. Duff & Phelps also provides professional services to the Bill & Melinda Gates Foundation and is likewise compensated for these services. As stated below, the Bill & Melinda Gates Foundation compensated Duff & Phelps under a broader engagement for the time spent on this manuscript. The authors themselves have not received any payments and declare no conflicts of interest.

## Role of the funding source

This work was funded by the Bill & Melinda Gates Foundation [OPP1114406]. The views expressed herein are solely those of the authors and do not necessarily reflect the views of the Foundation. The Foundation played a role in the generation of the research concept, but played no other role.
