# Supplementary material for: Cost of goods sold and total cost of delivery for oral and parenteral vaccine packaging formats
Source: Vaccine. 2018 Mar 14;36(12):1700–9. doi: 10.1016/j.vaccine.2018.01.011 (PMC5844852; doi:10.1016/j.vaccine.2018.01.011)
Supplement: Supplementary data 1 [file mmc1.docx]

# Supplementary material

## Appendix A: Cost of goods sold methodology

The analysis for packaging presentations was from a manufacturer’s perspective and based on an annualized cost build-up approach performed in Excel (Microsoft Corporation, Redmond, WA, USA). An annual period during a steady state of production was used in order to control for temporal variability in costs such as upfront capital costs or costs associated with lower manufacturing efficiencies at the startup period. Costs incurred by a manufacturer from postformulation through tertiary packaging were included; they were referred to as “fill-finish cost of goods sold (COGS).” Fill-finish COGS included: 1) facilities and equipment–related expenses, 2) raw materials and consumable expenses, 3) direct labor expenses, and 4) overfill and yield loss. The additional cost of 1) bulk formulation and 2) the delivery device (while not incurred by a manufacturer per se) was included in cases where this would be required for administration. We referred to the fill-finish COGS plus these additional costs as “COGS.” COGS (also referred to herein as “vaccine costs”) were a component of total cost of delivery (TCOD).

For oral vaccine packaging presentations, we evaluated blow-fill-seal (BFS) multi-monodose (MMD) ampoules, preformed polymer tubes, a single-dose glass vial, and a ten-dose glass vial. For parenteral vaccine packaging presentations, we evaluated a BFS MMD ampoule that would require a separate needle and syringe for delivery, a BFS compact prefilled auto-disable (CPAD) device with a separately packaged custom needle assembly, a preformed polymer CPAD device with an integrated needle, a single-dose glass vial, and a ten-dose glass vial. The BFS MMD ampoules for oral and parenteral vaccines were of a similar design, except for their volume and overfill capacity. The BFS CPAD device assumed five single-dose containers joined together, with each BFS CPAD container to be removed from the tab and attached to a needle assembly. The preformed polymer CPAD device had an integrated needle. Single-dose glass vials (oral and parenteral) were 2R/31 mm, and ten-dose oral and parenteral glass vials were 20R and 4R, respectively.

This analysis also explicitly excluded any upfront or ongoing product development costs or regulatory approval costs related to either the vaccine and/or the packaging presentation. Any profit margin assigned to the finished product was also excluded from the model due to high variability in margins and proprietary pricing.

The sections below provide the detailed methodology used to estimate the main cost components. Costs are ultimately estimated on a per dose basis.

1. **Facilities and equipment–related expenses** comprised depreciation, as well as annual ongoing facility overhead costs (e.g., repairs, maintenance, utilities). Depreciation is the annualized cost of the facility and equipment required to fill and finish 50 million doses of the same vaccine annually. The useful life was assumed to be 20 years for facilities and 10 years for equipment. Depreciation was calculated on a straight-line basis.

$$Facility \& equipment depreciation=$$

$$\frac{Capital expenditures}{Useful life}$$

Estimates of the capital expenditures for each presentation assumed an existing facility in the United States was retrofitted (i.e., a “brownfield” scenario). Square footage estimates were based primarily on the size of the requisite equipment and auxiliary space required by clean room grade. Cost estimates per square foot were segmented by grade of clean room. Class A was estimated at $1,750/ft^2^, Class B at $1,500/ft^2^, Class C at $1,000/ft^2^, and Class D at $750/ft^2^. Unclassified common space was held constant across packaging presentations and was estimated to cost $450/ft^2^. Cold storage construction costs were based on dimensions of the final packaged presentation, assuming 2.5 months of inventory on hand for each presentation and a consistent cost estimate for each cubic foot ($250/ft^2^) of cold storage.

Equipment capital expenditures assumed the purchase of requisite new equipment to run one manufacturing line capable of producing 50 million doses of a single vaccine per year. Cost estimates for filling machines were provided by machine manufacturers, as well as industry experts. Equipment validation costs were assumed to be the same across all presentations. Capital expenditure in absolute dollars was also assumed to be the same across all presentations for leak detection, particulate inspection, and labeling and secondary packaging equipment. Presentation specific equipment was also accounted for (e.g., dehydrogenation tunnel for the glass vial presentations).

Peripheral facility equipment capital expenditures for computer systems, laboratory equipment, security, and warehousing (e.g., forklift) were assumed to be equal for all presentations.

Facility overhead was based on the estimate of annual operating expenses of the facility, including repairs, maintenance, and utilities. Facility and equipment depreciation was multiplied by a uniform factor of one-third as a proxy for annual repairs and maintenance. Utilities were estimated based on the cost for the hypothetical facility, assuming installation of utility delivery systems had already occurred. Facility overhead also included indirect labor, which was held constant across all packaging presentations.

$$Facility overhead=$$

$$[\left( Facility \& equipment depreciation \right) X \left( \frac{1}{3} \right)]+Utilities+Indirect labor$$

1. **Raw materials and consumable expenses** were based on an average of low and high estimates provided directly from conversations with various personnel involved in the manufacturing or purchasing of the item.

Presentation-specific primary container raw materials that were assumed to be purchased from third parties by presentation include:

- Low-density polyethylene plastic for BFS.
- Vial, stopper, seal, and vial cap for single- and ten-dose glass vials.
- Preformed plastic tubes.
- Preformed CPAD device with integrated needle.

Secondary and tertiary packaging raw materials included foil overwrap (for polymer parenteral presentations only), labels (e.g., vaccine vial monitors), and cartons (both secondary and tertiary). Label costs were shared across five doses for the BFS MMD ampoules (oral and parenteral) and the BFS CPAD (parenteral) devices (as the BFS CPAD devices were assumed to be of an MMD proposed configuration) with 5 conjoined devices. Label costs were shared across ten doses for the ten-dose glass vial. The quantity of foil overwrap and cartons were determined based on the size of the packaged product in cubic meters relative to the dimensions of the foil overwrap and cartons multiplied by the cost of the foil overwrap and carton.

BFS MMD ampoules (oral):

$$Raw materials and consumables=$$

$Primary container raw materials+ \frac{VVM label cost}{5}+\frac{Secondary carton raw materials}{50}$

Preformed plastic tubes:

$$Raw materials and consumables=$$

$Primary container raw materials+VVM label cost+\frac{Secondary carton raw materials}{50}$

BFS MMD ampoules (parenteral) and BFS CPAD device:

$$Raw materials and consumables=$$

$$Primary container raw materials+\frac{VVM label cost}{5}+Foil overwrap cost+\frac{Secondary carton raw materials}{50}$$

Preformed CPAD device:

$$Raw materials and consumables=$$

$$Primary container raw materials+VVM label cost+Foil overwrap cost+\frac{Secondary carton raw materials}{50}$$

Single-dose glass vials:

$$Raw materials and consumables=$$

$$Primary container raw materials+VVM label cost+\frac{Secondary carton raw materials}{50}$$

Ten-dose glass vials:

$$Raw materials and consumables=$$

$$Primary container raw materials+\frac{VVM label cost}{10}+\frac{Secondary carton raw materials}{500}$$

1. **Direct labor expenses** were considered in three subprocesses: fill line operators, fill line clearance (for cleaning machines in between batches), and packaging line operators. Costs were based on hourly labor rates and the time required for filling 50 million doses, assuming a 500 L batch size for rotavirus vaccine (2.0 mL per dose) and a 125 L batch size for inactivated polio vaccine (IPV) (0.5 mL per dose). Calculations of the costs for each were based on the following:

*Fill line operators:*

*= # of fill line operators X filling hours per year X cost per hour, where*

*Filling hours per year:*

*= (# of containers per batch/effective speed) X batches per year, where*

*Effective speed:*

*= theoretical speed per hour X availability (i.e., numbers of hours machine can run adjusted for maintenance and downtime/hours in a year) X performance (i.e., time machine spends running correctly/total run time) X quality (i.e., time machine manufactures viable product/total time running), and*

*# of containers per batch:*

*= batch size (mL)/(dosage (mL) + overfill (mL) X quality X performance*

*Fill line clearance:*

*= # of line clearance employees X hours per changeover X cost per hour*

*Packaging line operators:*

*= # of packaging hours per batch X batches per year X cost per hour, where*

*# of packaging hours per batch:*

*= # of containers per batch/packaging speed per hour*

Total annual costs were then divided by annual production volume in all cases.

Annual volume calculations assumed a 2.0 mL oral dose and 0.5 mL parenteral dose.

1. **Overfill** ranges were constructed for each presentation to test the sensitivity of this input. For those presentations not being drawn from the container using a syringe, minimum overfill volumes were estimated based on a dose expression study conducted by PATH. Maximum overfill estimates were sourced from USP guidance [18]. For those packing formats requiring the dose be drawn from the container via syringe prior to delivery, the maximum and minimum overfill amounts are the same, as per the USP guidance.

Minimum and maximum overfill estimates by presentation are as follows:

| **Oral**  **Presentations** | **Minimum**  **Overfill%** | **Maximum**  **Overfill %** |
| --- | --- | --- |
| BFS Ampoules | 5% | 8% |
| Preformed Tube | 5% | 8% |
| Glass Vial—1-Dose | 8% | 8% |
| Glass Vial—10-Dose | 2% | 2% |

| **Parenteral**  **Presentations** | **Minimum**  **Overfill%** | **Maximum**  **Overfill %** |
| --- | --- | --- |
| BFS Ampoules | 20% | 20% |
| BFS CPAD | 11% | 20% |
| Preformed CPAD | 12% | 20% |
| Glass Vial—1-Dose | 20% | 20% |
| Glass Vial—10-Dose | 6% | 6% |

1. **Yield loss** was factored in as the estimated bulk product loss during production, based on the specific filling and finishing process for each product.

*Overfill cost per dose:*

*= bulk cost per dose X overfill percentage/dosage*

*Yield loss per dose:*

*= bulk cost per dose X (1–yield loss)*

1. **Delivery device** included the cost of a separately packaged syringe for the parenteral BFS ampoule, BFS CPAD device, and glass vial presentations. The cost of an oral delivery syringe (one per dose) and vial adapter (one per vial) was included for oral vial presentations. The cost of delivery devices was based on published prices in the United Nations Children's Fund (UNICEF) Supply Division catalog. For the oral presentations, the BFS MMD ampoules and preformed plastic tubes also served as the delivery device and did not require additional delivery supplies. The single-dose glass vials and ten-dose glass vials required vial adapters ($0.08 per vial) and oral delivery syringes ($0.04 per dose). The single vial adapter was shared across all ten doses for the ten-dose glass vials, while each dose required an individual oral syringe. For the parenteral vaccines, the preformed CPAD device was vendor purchased and included an integrated needle; as such, additional delivery supplies were not required. A 0.5ml auto-disable syringe ($0.04) was required for administration of each dose from a BFS MMD ampoule, single-dose glass vial, or ten-dose glass vial. The BFS CPAD device was assumed to feature a needle with a needle shield, which was separately packaged ($0.04).

Oral glass vials (single-dose and ten-dose):

$$Delivery device cost=$$

$$\frac{Oral syringe adapter cost}{number of doses per vial}+oral syringe cost$$

BFS CPAD device:

$$Delivery device cost=$$

$$propriatary needle assembly cost$$

BFS MMD ampoules and glass vials (single-dose and ten-dose):

$$Delivery device cost=$$

$$autodisable syringe cost$$

1. **Bulk formulation** cost was not directly calculated but was based on industry experts’ assessment of manufacturing costs. The cost of the bulk rotavirus vaccine was assumed to be $0.48 per dose. The cost of the bulk IPV was assumed to be $0.99 per dose.

## Appendix B: Total cost of delivery methodology

### In-country costs

The analysis was done using Excel (Microsoft Corporation, Redmond, WA USA). The outputs from the COGS analysis served as an input into the cost of delivery model. The output of the COGS analysis excluded profit margin and was not representative of an actual purchase price for the vaccine. Including a profit margin in the pricing would not affect our comparative analysis and would be difficult to accurately estimate given the highly variable nature of this factor between vaccine manufacturers and purchaser pairings.

1. Our analysis of the TCOD estimated costs for one year and one birth cohort from the point of receipt in country through the point of immunization delivery, using Kenya as a representative country. The model was based on data from Kenya’s 2011 comprehensive multiyear plan (cMYP) for immunization and published articles [18, 19, 20]. TCOD cost categories included: 1) vaccine cost, 2) transportation and storage, 3) administration, and 4) waste disposal. **Vaccine costs** were calculated as described above and were the output of the COGS analysis, which included the cost of the packaged vaccine and delivery device (if required). This cost was then applied to the number of doses required to vaccinate the target population in a given year. Programmatic vaccine wastage included both closed- and open-vial wastage. For oral rotavirus vaccine, which lacks preservatives, wastage rates were assumed to be 50% for ten-dose glass vials and 5% for all single-dose presentations (PATH estimates). For IPV, which contains preservatives, wastage rates were assumed to be 15% for ten-dose glass vials and 5% for all single-dose presentations (PATH estimates).

The quantity of vaccines required for one birth cohort for each presentation were estimated for each vaccine using the formula below:

$$Number of doses needed=$$

$$Population \times Surviving infant rate \times Vaccine target coverage rate\times\# of doses in schedule \times\frac{1}{1-Wastage rate}$$

For the sensitivity analysis of overfill’s impact on the TCOD, the vaccine cost outputs from the COGS sensitivity analysis on overfill were used.

1. **Transportation and cold chain storage costs** were associated with temperature-controlled transport and storage of the vaccine from the point of entry into Kenya’s cold chain to final point of use. Trucks, fuel, cold boxes, and average distance between cold chain facilities were used to cost transport of vaccines. Storage costs were calculated as a cost per liter of storage, including maintenance at each cold chain level based on existing cold chain equipment.

Using the number of doses required per year calculated above for each vaccine and presentation, we calculated the costs for cold chain storage and transportation. This was calculated by taking the cubic centimeter (cm^3^) volume of secondary packaging per dose for each presentation and applying this to the transport and cold chain structure in Kenya. Transportation costs were estimated by applying average distances between cold chain storage levels (national, regional, district, and health facility) and then applying a transportation cost per cm^3^ per kilometer. This cost per cm^3^ accounts for the cost of transportation equipment needed (trucks, cold boxes) and fuel costs.

Similarly, for cold chain storage the model used the current installed base of cold chain equipment in Kenya and created a weighted average cost per cm^3^ for each cold chain level. This included annualized cold chain equipment, energy, and maintenance costs.

Vaccines were delivered throughout the year at regular intervals. Cost and volume needs per supply interval were calculated for the storage and transportation by cold chain level.

$$Cold chain volume per level per supply interval=$$

$$\frac{Total doses needed \times Volume per dose \left( {cm}^{3} \right)}{\# of deliveries per year (by level)}$$

$$Transport cost per cm^{3}per km=$$

$$\frac{Annual capital cost + Fuel cost}{Average distance per year \left( km \right) \times Liters transported per trip \left( L \right)\times1000 \left( L/{cm^{3}} \right)}$$

$$Cold chain storage cost per cm^{3}=$$

$$\frac{Annualized Capex+Annual energy cost+Annual maintenance cost}{Equipment capacity (cm^{3})}$$

For the sensitivity analysis on packaging volume, we assumed a doubling in secondary volume, as compared to the primary analysis, for all of the polymer primary container packaging formats. This degree of variability was determined based on the potential for variability in primary container design and the potential for primary containers to not be nested within the secondary packaging, which is also dependent on the container design.

Glass vials are of a standardized design and have limited options for secondary packaging layouts; as such, we assumed that packaged volume for glass vials for both oral and parenteral vaccines did not change.

For those packaging formats requiring a separately packaged delivery device (glass vials—both oral and parenteral, BFS MMD ampoules—parenteral and BFS CPAD), the packaged volume of the delivery device itself was assumed to not change.

Packaged volumes for the sensitivity analysis are as follows:

|  | **Oral Vaccine Presentations** | | | | | | | |
| --- | --- | --- | --- | --- | --- | --- | --- | --- |
|  | **BFS MMD**  **ampoules** | | **Preformed**  **polymer tubes** | | **Single-dose**  **glass vials** | | **Ten-dose**  **glass vials** | |
|  | **Min** | **Max** | **Min** | **Max** | **Min** | **Max** | **Min** | **Max** |
| **Packaged vol./dose (incl. 2° packaging)** | 9.4 cm^3^ | 18.8 cm^3^ | 13.4 cm^3^ | 26.8 cm^3^ | 8.0 cm^3^ | | 4.8 cm^3^ | |
| **Delivery device volume** | - | | - | | 36.5 cm^3^ | | 31.1 cm^3^ | |
| **Waste disposal vol./dose** | 9.4 cm^3^ | 18.8 cm^3^ | 13.4 cm^3^ | 26.8 cm^3^ | 44.5 cm^3^ | | 35.9 cm^3^ | |

|  | **Parenteral Vaccine Presentations** | | | | | | | | | |
| --- | --- | --- | --- | --- | --- | --- | --- | --- | --- | --- |
|  | **BFS MMD ampoules** | | **BFS CPAD device** | | **Preformed CPAD device** | | **Single-dose glass vial** | | **Ten-dose glass vial** | |
|  | **Min** | **Max** | **Min** | **Max** | **Min** | **Max** | **Min** | **Max** | **Min** | **Max** |
| **Packaged vol./dose (incl. 2° packaging** | 7.8 cm^3^ | 15.6 cm^3^ | 7.6 cm^3^ | 15.2 cm^3^ | 10.5 cm^3^ | 21 cm^3^ | 8 cm^3^ | | 1.1 cm^3^ | |
| **Delivery device volume** | 42.8 cm^3^ | | 16 cm^3^ | | - | | 42.8 cm^3^ | | 42.8 cm^3^ | |
| **Waste disposal vol./dose** | 50.6 cm^3^ | 58.4 cm^3^ | 23.6 cm^3^ | 31.2 cm^3^ | 10.5 cm^3^ | 21 cm^3^ | 50.8 cm^3^ | | 43.9 cm^3^ | |

1. **Administration** included the cost of a health care worker’s time required for vaccine administration in the routine immunization setting. This cost was calculated by taking the time required for a health care worker to open vaccine packaging, draw vaccine from the vial or ampoule (as appropriate), and administer vaccine to the infant (orally or by injection). Administration times for the existing presentations were calculated using published and unpublished PATH time-motion studies, and then costed by applying salary rates for nurses in Kenya obtained from the cMYP [18, 21]. Administration times for novel delivery technologies were based on target product profile guidance documents, internal PATH assessments, and correlation to previously conducted studies [21]. Costs and time for other tasks conducted during the infants’ immunization visit, which were not anticipated to vary between presentations, were not included.

For each technology, the cost of health worker time for vaccine administration was estimated as:

$$Cost to administer a dose=$$

$$Time to administer vaccine \left( seconds \right) by technology\times Health care worker salary \left( {\$}/{Second} \right)$$

1. **Waste disposal costs** included the disposal of the vaccine packaging, delivery technology, and supplies in a safety box. Disposal costs were calculated by assuming all facilities used a 5 L safety box, then calculating the number of doses (vial or BFS packaging, needle, and syringe as applicable) that would fit in each box based on the volume per dose. We then calculated the total number of safety boxes needed for the birth cohort for each presentation based on the vaccine demand estimated and multiplied this by the cost of a safety box. Safety box cost was estimated at $0.48 per box (UNICEF Supply Division average) and cost of disposal was estimated to be $1.00 per safety box (PATH estimate of a full safety box weighing 1 kg and a $1/kg disposal cost).

$$\# of doses fitting into a safety box=$$

$$\frac{5000 {cm}^{3} of safety box volume}{Volume of waste per dose per technology (cm^{3})}$$

$$\# of safety boxes needed=$$

$$\frac{Target \# of doses needed}{\# of doses fitting into a safety box}$$

$$Total disposal cost=$$

$$(\# of safety boxes needed \times Cost per safety box)+(\# of safety boxes needed \times Cost of disposal)$$

In the sensitivity analysis, the same range of container volumes that were evaluated for transport and storage cost impact were also assessed for impact on disposal costs.
